# Addressing unmet basic needs for children with sickle cell disease in the United States: clinic and staff perspectives

**DOI:** 10.1186/s12913-020-06055-y

**Published:** 2021-01-12

**Authors:** Stephanie Loo, Annelise Brochier, Mikayla Gordon Wexler, Kristin Long, Patricia L. Kavanagh, Arvin Garg, Mari-Lynn Drainoni

**Affiliations:** 1 Department of Health Law, Policy and Management, Boston University School of Public Health, 715 Albany Street, Boston, MA 02118 USA; 2 Department of Pediatrics, Boston Medical Center, Boston, USA; 3 Department of Psychological and Brain Sciences, Boston University, Boston, USA; 4 Boston University School of Medicine, Boston, USA; 5 Section of Infectious Diseases, Department of Medicine, Boston University School of Medicine, Boston, USA; 6 Evans Center for Implementation and Improvement Sciences, Boston University, Boston, USA; 7 Center for Healthcare Organization and Implementation Research, ENRM Veteran’s Administration Hospital, Boston, USA

**Keywords:** Social determinants of health, Unmet basic needs, Pediatric hematology, Sickle cell disease, Clinical staff perceptions

## Abstract

**Background:**

The purpose of this study was to assess pediatric hematology clinic staff’s perspectives regarding barriers and facilitators in addressing unmet basic needs for children with sickle cell disease (SCD).

**Methodology:**

Six focus groups were held at four urban pediatric hematology clinics in the Northeastern region of the United States from November to December 2019. Discussion questions were developed to align with the integrated Promoting Action on Research Implementation in Health Services (i-PARIHS) implementation science framework, focusing on the domains of context and recipient and how clinics address adverse social determinants of health (SDoH) in their patient populations. A summative content analytical approach was taken to identify major themes in the data.

**Results:**

We discerned the following themes: (1) families of children with SCD experience numerous unmet basic needs; (2) clinic staff believed they had a role to play in addressing these unmet basic needs; (3) staff felt their ability to address families’ unmet basic needs depended upon caregivers’ capacity to act on staff’s recommendations; and (4) clinic staff’s ability to address these needs was limited by organizational and systemic factors beyond their control.

**Conclusions:**

These findings have important implications for how best to address adverse SDoH for this vulnerable pediatric population so that urban-based pediatric hematology clinics can more equitably support families.

**Supplementary Information:**

The online version contains supplementary material available at 10.1186/s12913-020-06055-y.

## Background

The environments in which children are born, grow, work, live, and age—understood collectively as social determinants of health (SDoH)—are key drivers of health and wellbeing [1]. The field of pediatrics has led the charge for addressing SDoH, specifically unmet basic needs (such as food, housing, and utilities insecurity) which predispose low-income children to adverse health outcomes. American Academy of Pediatrics guidelines encourage all pediatricians to screen families for unmet basic needs, connect families with identified needs to resources in the community, and cultivate medical homes where care is family centered [2]. These guidelines further acknowledge the pervasiveness of race-based health inequities, which while most evident in urban areas affected by residential segregation and environmental racism, have become entrenched in suburban and rural America over the last decade [2].

Unmet basic needs may be even more prevalent among children with chronic health conditions than in the general pediatric population and can directly (through exposure to toxic stress, thereby altering physiological pathways) [3] or indirectly (through impeding access to treatment) exacerbate their underlying condition. Sickle cell disease (SCD) is an inherited red blood cell disorder that affects approximately 36,000 [4] children in the United States, of whom more than 80% identify as ‘Black.’ This population is disproportionately impoverished in all areas that impact health compared to non-Hispanic White Americans [5]. One study conducted in a United States-based urban pediatric hematology clinic found that over 90% of children with SCD had at least one unmet basic need [6]. Children with SCD can have episodes of severe pain, are at greater risk of sepsis and stroke, and have significantly shorter life expectancies compared to their peers [7]. As such, children with SCD often have frequent points of contact with specialty care providers who support patients and their families in managing the medical and psychosocial aspects of the disease.

In response to this, a multitude of screening and referral models for addressing families’ unmet basic needs have emerged in urban pediatric primary care clinics [8]. These sites serve as critical settings for such interventions given their typically centralized locations and capacity to have the resources to serve broad catchment areas. Yet, despite the demonstrated needs of the patient population [6, 9], similar SDoH screening and referral models have not been as widely investigated or implemented in pediatric subspecialty care. Our study addresses this significant gap in the literature by assessing urban pediatric hematology clinic staff’s perspectives regarding barriers and facilitators to addressing unmet basic needs experienced by families with a child with SCD.

## Methods

### Setting

In May 2019, four urban pediatric hematology clinics in the Northeastern region of the United States were invited and agreed to participate in a pilot study to implement a screening and referral intervention for unmet basic needs. This manuscript describes qualitative work aimed at understanding clinic practice prior to implementing the intervention. The study was approved by the Boston Children’s Hospital Institutional Review Board and informed consent was obtained from each participant.

Participating clinics served 220 patients on average (range: 130–300) from a wide catchment area—including rural and suburban residing populations. Each clinic had one or two dedicated social workers who conducted standardized psychosocial assessments with patients and acted as liaisons between patients’ families and community resources; however, none of the clinics had a systematic approach for identifying and addressing unmet basic needs.

### Recruitment and data collection

Recruitment emails were sent to clinic staff providing direct care to SCD patients at the four sites, inviting them to participate in focus group discussions. Six focus groups were held at times convenient for participants. The study team provided refreshments, but participants received no other compensation. Focus groups were led by a single facilitator experienced in qualitative research, while two additional study staff observed and recorded field notes. With participant permission, focus groups were audio-recorded. Recordings were professionally transcribed verbatim, and transcripts were de-identified and reviewed for accuracy by study staff.

The integrated Promoting Action on Research Implementation in Health Services (i-PARIHS) framework [10] was used to inform and develop a focus group question guide (Supplementary File 1). The i-PARIHS framework presents implementation as a function of an innovation and its evidence base (both theoretical and experiential knowledge), recipients (those who are affected by and who influence implementation), and contextual factors internal and external to the environment in which the innovation is implemented. Accordingly, key areas of exploration in these focus groups were staff’s past experiences and current practices for addressing unmet basic needs; staff’s attitudes about their role in addressing patients’ unmet basic needs; and clinic-, organization-, and systems-level contextual factors that shape staff’s current practices and beliefs (Table 1).

**Table 1:** Sample Questions from Focus Group Discussion Guide

i-PARIHS Construct	Sample Questions
***Innovation***	What is your clinic’s current standard of care regarding addressing unmet basic needs such as food and housing for your patients?
	How appropriate is this standard?
***Recipients***	In general, what do you see as the role of addressing unmet basic needs in SCD clinics?
	Why do you see having a standard practice for addressing unmet basic needs as important or unimportant?
	What would this standard practice look like to you?
	Would such a standard practice be feasible in your clinic?
***Context***	How important is addressing patients’ unmet basic needs to your mission?
	In general, what is it like trying to adopt a new program or intervention in your clinic?
	What is usually the response from program leadership?
	What are the major contextual factors you have to address?
***Facilitation***	*N/A for pre-implementation focus groups*

### Data analysis

A summative content analytical approach was taken using the i-PARIHS constructs of context, innovation, and recipient to develop the initial codebook structure [11]. Two transcripts were initially analyzed by a three-person coding team (SL, AB, MGW) to determine alignment of the focus groups with the thematic framework. Each member of the coding team then independently coded the remaining four transcripts, with all transcripts coded in triplicate. The coding team met weekly to review individual coding of each transcript and come to consensus on any discrepancies between coders, supervised by the qualitative and implementation science expert (MD). Findings were reviewed and agreed upon by the entire research team.

## Results

Six focus groups were conducted between November and December 2019 (*N* = 46) with the number of participants ranging from 4 to 10. The majority of participants were female (89%), aged 45–54 years (37%), non-Latinx (87%), and Caucasian (84%), and had worked in the SCD clinic an average of 7.5 years. Participants included nurses, medical providers (physicians, nurse practitioners, and physician assistants), psychosocial providers (psychologists and social workers), pediatric hematology research staff, and clinic support staff (Table 2).

**Table 2:** Sociodemographic Characteristics of Focus Group Participants (*N* = 46)

**Gender, N (%)**	
Female	41 (89.1)
Male	5 (10.9)
**Age, years, N (%)**	
18–24	2 (4.3)
25–34	7 (15.2)
35–44	12 (26.1)
45–54	17 (37.0)
55–64	4 (8.7)
65–74	4 (8.7)
**Race, N (%)**	
Asian	1 (2.2)
Black or African American	5 (11.1)
Caucasian or White	38 (84.4)
Other	2 (4.4)
**Ethnicity, N (%)**	
Hispanic, Latino/a/x, or Spanish	6 (13.0)
Not Hispanic, Latino/a/x, or Spanish	40 (87.0)
**Clinic Role, N (%)**	
Nurse	10 (21.7)
Psychosocial provider	11 (23.9)
Medical Provider	14 (30.4)
Front desk or support staff	6 (13.0)
Site research staff	5 (10.9)
**Years worked in clinic, median (range)**	7.5 years (1 month-30 years)

Through our analysis, we found four overarching themes: (1) families of children with SCD experience numerous unmet basic needs; (2) clinic staff believed they had a role to play in addressing unmet basic needs of patients and their families; (3) staff felt their ability to address families’ unmet basic needs depended upon caregivers’ capacity to act on staff’s recommendations; and (4) clinic staff’s ability to address unmet basic needs was limited by organizational and systemic factors beyond their control. While each quotation below presents the viewpoint of individual participants, we carefully selected quotations reflecting perspectives shared consistently across all four sites.

### Families of children with SCD experience numerous unmet basic needs

Staff agreed that unmet basic needs were pervasive among their patients with SCD, relative to other patient populations seen within their hospitals.

*Psychosocial provider: “I'll just put it out there, the need in this particular population is great, in general, by ratio … I think there's a good number of those that would have issues with insecurities around the issues [food, housing, etc.] that you're talking about.”*

Due to frequent visits, needs were often reported to staff without prompting. Staff cited an array of common needs experienced by patients and their families, including limited access to warm clothing, housing and nutritious food.

*Nurse: “Usually you can tell [food insecurity] by the kid too. We have kids coming in and we give them a juice box and it's like they haven't drank for three days so...it's kind of a little red flag.”*

Clinic staff expressed that, though unmet basic needs were common in their communities, the impact of unmet basic needs on patients with SCD is particularly severe given their medical complexity. Staff across all clinics considered utility protection letters^1^ to be critically important, as experiencing cold temperatures due to heat shut-off can trigger vaso-occlusive pain.

*Psychosocial provider: “We do a lot of those...utility shutoff protection letters. I get asked almost daily for one of those from one of our patients. They have bills that are probably in the thousands.”*

Additionally, transportation difficulties impacted SCD patients and their families, as inability to travel between home and clinic limited their access to care and increased barriers to obtaining medication to manage their SCD.

*Medical provider:* ***“****[A] lot of our younger children … are on hydroxyurea. It's a medication to treat sickle cell... There's not a lot of pharmacies that compound it. I have one family that's driving up from [city] every month to [city], driving and parking here to pick up a prescription.”*

### Clinic staff believed they had a role to play in addressing unmet basic needs of patients and their families

Though clinics’ practices for addressing unmet basic needs were not uniform, all clinics employed at least one part-time social worker and acknowledged the link between medical and basic needs for children with SCD. Staff believed they had fostered trust and rapport with families, enabling families to feel comfortable disclosing their needs.

*Medical provider: “I know that when I started in my position... the one thing that I demanded was a social worker..., [as] I knew that I wasn't going to be able to improve the health and wellbeing of these kids if I didn't have my own social worker.”*

*Psychosocial provider:* ***“****We meet [with patients] at every appointment. And they might be fine for a couple years, and then they might have a psychosocial need. But then we've established rapport with them, we know their story.”*

Despite underscoring the importance of addressing unmet basic needs within SCD clinics, staff also noted that attempting to address these needs within complex medical visits cannot rest solely on social workers. Staff felt limited in their abilities to proactively address patients’ needs, given that demand for assistance far outpaced the capacity they had in their roles.

*Psychosocial provider:* ***“****[T] he medical issue is obviously the priority, that's why they are here, but you can't ignore all the other aspects of their life which inevitably impact their medical situation. They come once every six months and then we may have identified six social issues or one social issue. And the ability to sort of help carry that through and solve those problems with them—I think it's super challenging for social work and that's where I think the burden falls.”*

### Staff felt their ability to address families’ unmet basic needs depended upon caregivers’ capacity to act on staff’s recommendations

Clinic staff described their perceptions of how caregivers understand the role of SCD clinics in addressing unmet basic needs. Through frequent visits required to manage children with SCD and the strong rapport clinic staff felt they had developed with families during these interactions, staff believed that caregivers perceived the pediatric hematology clinic as an accessible support system to address their unmet basic needs. Some providers suggested caregivers had come to expect help with non-clinical needs.

*Medical provider: “I think it's sort of sometimes very hard to judge whether it's a need or it has become a habit. I think some families, it may not be a need because, I don't want to put every sickle cell family in the same category that it's a need, but I think it becomes a habit.”*

Staff suggested that when they offered information about external resources, they were uncertain if caregivers had the time or knowledge necessary to connect to resources outside of the hospital.

*Psychosocial provider****: “****And I think the other issue is they [the families] haven't accessed those things because they don't know how to do it or they don't know what to do. And then you give them [phone] numbers, and I'm not saying everybody, but oftentimes there's a breakdown that you give them numbers and then the follow through is just lacking.”*

Staff described the difficulty of balancing their desire to help families address unmet basic needs while supporting caregivers’ capacity-building. Staff felt compelled to help with resources on behalf of families, which oftentimes required substantial investment of time. Clinic staff voiced concern that their actions may diminish caregivers’ roles in self-advocating and problem-solving.

*Psychosocial provider:* ***“****So you know, sometimes we can have this culture of giving, which is wonderful. But also it can sometimes not be helpful for the families in terms of helping them sort of problem-solve on their own or seek resources. It's a fine balance between that.”*

### Clinic staff’s ability to address unmet basic needs was limited by organizational and systemic factors beyond their control

Staff expressed frustration about the unequal distribution of resources across subspecialty clinics within their organizations. Clinics were jointly housed with oncology clinics, and staff noted a contrast between the resource-rich pediatric oncology services and the limited resources available for their patients with SCD.

*Psychosocial provider: “I wish that there were just more sickle cell resources...it's frustrating...especially when you share a section with oncology [that] is so rich in resources everywhere you look. And then sickle cell there's nothing and that's not an exaggeration … that's hard.”*

In addition, staff described how organizational structures and hospital restrictions limited the resources they were able to provide patients to address basic needs.

*Psychosocial provider: “I wish I could give out gift cards for grocery stores and gasoline. Our hospital has more recently had this strict aversion based on corporate compliance and we can't give out gift cards.”*

Our findings also considered the broader political, legal, and health systems in which each clinic operates. Staff noted barriers they encountered when navigating a complex healthcare system in a challenging political climate.

*Psychosocial provider: “We are the catchment area for [city]. We take everybody, which is wonderful..., but trying to help them navigate the healthcare system can be very complex. Especially if they're immigrants and English is a second language. There's a lot of education involved [in] trying to connect them with resources.”*

Some providers expressed that though they had a role to play in addressing unmet basic needs, they questioned their ability to intervene on issues between patients and systems outside the hospital’s domain such as health insurance, public housing, or patients’ immigration status.

*Medical provider: “[We] can direct them to resources available, [but]...can we really fix this as a medical community? This is really more a government policy issue and what we can do is direct them to resources. Give them addresses of food pantries, give them addresses or give them resources of how to contact the housing department to put them on the list for assisted housing. But I think this is...more a broader government and policy issue.”*

## Discussion

This study uniquely explores barriers and facilitators to addressing unmet basic needs in the urban United States pediatric subspecialty setting. Our results suggest that SCD clinics believe it is vital to address unmet needs (e.g., food insecurity, letter of medical necessity to prevent heat shut-off) but that there are important barriers at the family, clinic, and societal levels that impede clinic staff’s ability to address these needs.

Staff described significant unmet basic needs among their SCD patients and expressed that their capacity to address needs while fulfilling clinical responsibilities was strained. Studies evaluating initiatives in safety-net hospitals reveal greatest success occurs when providers feel they have adequate organizational support and sufficient time and resources to fulfill multiple roles [13]. Potential solutions for streamlining SCD clinic staff’s efforts could include systematic SDoH screening and referral interventions and embedding community health workers (CHWs) within clinic teams. Screening and referral interventions standardize the process for identifying unmet basic needs and connecting families to needed services. Such interventions have been shown to increase caregiver enrollment in community-based resources within pediatric primary care [8]. CHWs could aid caregivers in accessing resources referred by clinic staff [14–20], though funding for CHWs would require buy-in at the organizational level. Federal agencies are recognizing the importance of this role, and are providing training for CHWs in a variety of disease-specific clinical settings, including HIV and SCD clinics [21, 22].

In our study, staff acknowledged that clinic- and organization-level change is necessary to adequately address SCD patients’ basic needs, yet they felt their capacity to mitigate unmet basic needs was limited by systemic factors beyond their control. Medical-legal partnerships, whereby attorneys provide families with legal advice and representation on issues such as housing, immigration, and education services, has been used in pediatric primary care. Introducing medical-legal partnerships into pediatric SCD care could facilitate the systems-level change that is so urgently needed [23].

Some staff questioned caregivers’ capacity to connect to resources outside of the clinic. Critically, this occludes the fact that families in need of resources may have little support, time, or social capital to identify and connect with these services independently [24, 25]. Caregivers’ “habit” of accessing support for basic needs through the SCD clinic speaks to a larger societal problem of systemic inequity and lack of a sufficient safety net that makes navigating services difficult—a process further exacerbated by managing a complex chronic illness. Assumptions about caregivers’ lack of self-sufficiency are contradicted by research in an urban pediatric hematology clinic, which demonstrated that 45% of families provided with a community referral had communicated with the community agency within 2 weeks of receiving the referral [9]. However, when help is offered, families may be reluctant to accept. In a study of caregivers’ perspectives on provider assistance with community services, caregivers expressed feeling judged by those providing services, which compromised their motivation to contact and enroll in resources [26]. Further investigation is needed to determine the attitudes of families and the barriers they face in accessing community resources.

Medical clinics, especially those working with underserved populations, are not sufficiently resourced to substitute for a more robust social services system [27, 28]. The divergence between clinic expectations versus capacity is compounded by implicit biases that may have manifested, since the majority of SCD providers differ in race or socioeconomic background from their patients [29]. While this initial qualitative work did not include formal implicit bias assessments of participants [30], care should be taken regarding potential unconscious bias and attitudes during the delivery of clinical care services [31, 32]. The use of a strength-based approach in provider-family communication is necessary and would better equip clinic staff to recognize the resiliency and capacity of caregivers as they navigate medical and social spheres [33]. Additionally, training health care providers on race and racism may increase awareness of the systemic barriers that face persons of color in the United States [34, 35].

Our study had several limitations. First, our sample size was restricted to four SCD clinics in one United States geographic region; our results may not be generalizable to pediatric hematology outpatient clinics in non-United States contexts, though research in international settings has reported on the efficacy of community approaches to SCD management [36–38]. Two qualitative studies, in Brazil and Benin, acknowledged the need for all-encompassing family and community support as best practices for the management of sickle cell disease [37, 39].

We chose to conduct focus group discussions because they offer a comprehensive yet time- and cost-efficient way to gather desired information [40]. We recognize that power dynamics within the group setting may have made some participants more reticent to share their views than if we had conducted individual key informant interviews; for instance, a medical assistant might have hesitated to voice criticism of a hematologist. Other SCD clinics may have different workflows and processes for addressing patients’ unmet basic needs than reported here. This study focused on clinic staff’s perceptions regarding their current practices for addressing basic needs and did not directly interview caregivers of SCD patients regarding their own perceptions of the clinic’s practices and role. Follow-up studies by this team will address SDoH issues from the caregiver perspective.

## Conclusion

Staff from urban pediatric hematology clinics acknowledged the pervasiveness of unmet basic needs among patients with SCD, while recognizing the role they can play in addressing needs in the face of under-resourced clinics and systemic barriers. In order to effectively care for patients with SCD and based on our focus groups with staff, these clinics should be equipped to address unmet basic needs over and above patients’ medical concerns. These findings have important implications for how best to address adverse SDoH for this vulnerable pediatric population so that pediatric hematology clinics can more equitably support families and provide social care as it is integral to health outcomes. This will likely require acknowledging the inequitable systems within which SCD clinics operate, and implementing tools and partnerships to systematically assess and address unmet basic needs.

## Supplementary Information

**Additional file 1.**

## Data Availability

The datasets used and/or analyzed during the current study are available from the corresponding author on reasonable request.

## Abbreviations

SDoH: Social determinants of health
SCD: Sickle cell disease
i-PARIHS: Integrated Promoting Action on Research Implementation in Health Services
CHW: Community health worker

## Footnotes

1 A utility protection letter is written by a healthcare provider to prevent shut-off of heating sources due to reasons of medical necessity (i.e., the symptoms of a child with SCD can be exacerbated by fluctuations in environmental temperature) [12].
