# Supplementary material for: Addressing unmet basic needs for children with sickle cell disease in the United States: clinic and staff perspectives
Source: BMC Health Serv Res. 2021 Jan 12;21:55. doi: 10.1186/s12913-020-06055-y (PMC7802171; doi:10.1186/s12913-020-06055-y)
Supplement: Supplementary file 1 — Additional file 1. [file 12913_2020_6055_MOESM1_ESM.docx]

**Sickle Cell disease WE CARE STUDY**

**Pre-implementation Focus group discussion guide**

**Example Questions & Probes from the i-PARIHS framework (addressing innovation, context, recipients, facilitation)**

1. What is your clinic’s current standard practice regarding addressing unmet social needs such as food and housing for patients?
   1. How do you address them, if at all?
      1. If not, why doesn’t your clinic address unmet social needs?
   2. What do you think about your clinic’s practice regarding this?
      1. In general, what do you see as the role of addressing social needs in hematology clinics?
   3. Are you interested in your clinic having a standard practice for addressing unmet social needs?
      1. Why do you see having a standard practice for this as important or unimportant?
      2. What would this standard practice look like to you?
         1. Would such a standard practice be feasible in your clinic?
2. What do you currently do if a patient’s family reports unmet social needs?
   1. How do you address them?
   2. Who tends to take responsibility for addressing them?
   3. How is the information about a patient’s unmet social needs typically communicated to you?
   4. Do you think that many of your patients or families have unmet social needs? Frequency?
      1. What kinds of unmet needs do your patients tend to have? Most common? Less of an issue?
3. What do you think about the appropriateness of your clinic providing community resource information for unmet social needs?
   1. How does this fit into your mission?
   2. What about when resource referrals are integrated with social needs screening?
   3. Who should provide them? (e.g., social worker, provider, navigator, other)
4. In general, in your clinic, what is it like trying to adopt a new program or intervention?
   1. How does it work? Who is involved?
   2. How easy or difficult is it to try new things?
   3. What is usually the response from program leadership?
   4. What are the major contextual factors you have to address (buy-in from organization, leadership in and outside of clinic, other internal or external contextual factors)
5. Are there any other important things we have not discussed?

*Thank you for your time. We will be back to speak with you after implementation has started to get your opinions.*
